# Multiple automated machine-learning prediction models for postoperative reintubation in patients with acute aortic dissection: a multicenter cohort study

**DOI:** 10.3389/fmed.2025.1531094

**Published:** 2025-04-11

**Authors:** Shuyu Wen, Chao Zhang, Junwei Zhang, Ying Zhou, Yin Xu, Minghui Xie, Jinchi Zhang, Zhu Zeng, Long Wu, Weihua Qiao, Xingjian Hu, Nianguo Dong

**Affiliations:** ^1^Department of Cardiovascular Surgery, Union Hospital, Tongji Medical College, Huazhong University of Science and Technology, Wuhan, Hubei, China; ^2^Hubei Cardiovascular Surgery Quality Control Center, Wuhan, China

**Keywords:** Type A aortic dissection, reintubation, machine learning, predictive model, SHAP value

## Abstract

**Introduction:**

Reintubation is an adverse postoperative complication in patients with Type A aortic dissection (AAD) that correlates to poor outcomes. This study aims to employ machine learning algorithms to establish a practical platform for the prediction of reintubation.

**Methods:**

A total of 861 patients diagnosed with AAD and undergoing surgical procedures, 688 patients as training and testing cohort from a single center, and 173 patients as validation cohort from four centers were enrolled. The least absolute shrinkage and selection operator (LASSO) was used for screening risk variables associated with reintubation for subsequent model construction. Subsequently, seven machine-learning models were built. The model with the best discrimination and calibration performance was used to predict reintubation. Finally, the SHapley Additive exPlanation (SHAP) was employed to explain the prediction model.

**Results:**

Reintubation was performed in 107 patients (12.43%). The LASSO analysis identified re-admission to the intensive care unit (ICU), continuous renal replacement therapy, length of stay in the ICU, and duration of invasive mechanical ventilation as significant risk factors for reintubation. The XGBoost model was selected as the final prediction model due to its better performance than other models, with the AUC, sensitivity, and specificity of 0.969, 0.8889, and 0.8611 in the testing cohort. SHAP values demonstrated the effects of individual features on the overall model. Finally, a web calculator was developed based on XGBoost model for the clinical use.

**Conclusion:**

We have developed and validated a high-performing risk prediction model for postoperative reintubation in patients with AAD. It can provide valuable guidance to clinicians in predicting reintubation and in developing timely preventative measures.

## 1 Introduction

Type A aortic dissection (AAD) is a devastating condition characterized by an entry tear in the aortic intima or hemorrhage in the aortic media, leading to the separation of the ascending aortic layers (1, 2). Due to the sudden onset and rapid progression of AAD, the mortality rate alarmingly increased by 1–2% per hour within the first 48 h after symptom onset, if not intervened promptly (3). Open surgery is a life-saving operation and remains the preferred treatment modality for patients with AAD (4). Advances in surgical treatments have substantially reduced the operative mortality from AAD, however, the postoperative in-hospital mortality rates remain relatively high, reported to be up to 25% (5, 6). Postoperative complications, including respiratory failure, stroke, new-onset renal failure, and neurological impairment are the main contributors to these high mortality rates (7). Consequently, timely prediction, identification, and management of postoperative complications is a valuable and critical task for improving patient prognosis.

Reintubation is a common complication after cardiac surgery, with an incidence ranging from 2 to 13% (8). Postoperative reintubation can lead to adverse patient outcomes, including increased postoperative blood transfusion requirements, acute kidney injury, delirium, prolonged length of stay in the intensive care unit (ICU LOS), and increased mortality rates (9, 10). Therefore, predicting the risk of reintubation is crucial for maximizing patient benefits and minimizing harm. A retrospective study included postoperative patients undergoing a variety of cardiac procedures, including aortic surgery, and identified 10 risk factors associated with postoperative reintubation, including older age, previous cardiac surgery, congestive heart failure, emergency surgery, and longer duration of surgery (11). Another study focusing on pediatric cardiac surgery has delineated three risk factors for reintubation, including patient characteristics, high complexity of surgery, and patients undergoing surgery at low-volume centers (12). Given the significant differences in patient characteristics and disease conditions across different types of cardiac surgery, it is necessary to analyze postoperative reintubation risk factors separately for different diseases or surgeries to avoid the interference of confounding factors. Although the incidence of reintubation in patients with AAD is relatively high in cardiac surgery (13–15), there is currently no specific research on the risk prediction of reintubation for AAD patients. Addressing this gap in the literature will help implement effective preventive strategies, thereby reducing the incidence of reintubation in AAD patients postoperatively.

Over the past decades, machine-learning has emerged as a promising method for data mining and analysis, widely applied as a predictive tool in clinical practices (16, 17). The predictive accuracy of machine-learning has been shown to outperform traditional statistical methods (18). Machine-learning has the ability to analyze vast amounts of data, uncover subtle predictive risk factors, and to improve the predictive power to enhance clinical guidance (19, 20). In addition, most traditional studies are restricted by small sample sizes, the use of complex system variables that are not easily accessible, and a lack of external validation, severely hampering the clinical applicability of these models. Exploring risk factors through machine-learning models based on multicenter data and constructing a feasible and efficient predictive model is expected to facilitate early identification and early intervention for postoperative reintubation in AAD patients, thereby improving patient prognosis.

Therefore, in the present study, we aim to develop and validate seven machine-learning models by incorporating diverse perioperative data, to predict reintubation in AAD postoperative patients based on multicenter data, and ultimately visualize the model with the best predictive to improve the usability of this predictive model.

## 2 Materials and methods

### 2.1 Study population and data partitioning

This is a multicenter, retrospective, observational cohort study. A total of 892 patients with AAD who underwent open surgery from five medical centers in China, including Wuhan Union Hospital, Xiangyang Central Hospital, Shiyan Taihe Hospital, Yichang Central Hospital, and Jingzhou Central Hospital, between January 2020 and October 2023 were enrolled. Inclusion criteria were as follows: (1) patients diagnosed with AAD admitted for open surgery; (2) aged 18 years or older. Exclusion criteria included: patients deceased during or within 24 h after surgery. This study was approved by The Ethics Committee of Tongji Medical College of Huazhong University of Science and Technology (IORG No. IORG0000521) under the declaration of Helsinki.

We developed prediction models based on the patient cohort from Wuhan Union Hospital, which comprised 688 patients with AAD who underwent open surgery. They were randomly assigned to training and testing cohorts in a 7:3 ratio. To further assess the performance of the models, data from the other four hospitals were used for subsequent external validation, which comprised 173 patients with AAD who underwent open surgery.

### 2.2 Data collection

Characteristics such as general demographic characteristics, medical history, preoperative laboratory test, intraoperative information, and postoperative information and laboratory test were acquired through the hospital’s electronic medical recording management system. The requirement for patient approval or written informed consent was waived due to the retrospective nature of this study. Preoperative data included in the analyses were age, gender, weight, height, body mass index (BMI), smoking history, alcohol usage history, pulmonary complications, hypertension, diabetes mellitus, Marfan syndrome, brain complications, renal insufficiency, previous surgery, and laboratory data. Surgery-related information and postoperative blood gas analysis results were also obtained. For continuous variables, missing values were imputed using the mean if the data were normally distributed or the median if the data were skewed. For categorical variables, missing values were replaced with the most frequent category. If the proportion of missing values for a variable exceeded 30% of the total observations, the variable was excluded from further analysis to ensure data reliability.

Body mass index is calculated by dividing weight in kilograms by the square of height in meters. Hypertension and its classification were based on the 2018 Chinese Guidelines for Prevention and Treatment of Hypertension (21). Diabetes mellitus was defined based on a previous diagnosis, use of diabetes medications, or random blood glucose ≥11.1 mmol/L or fasting blood glucose ≥7.0 mmol/L. Renal insufficiency was defined by a previous diagnosis or serum creatinine above 110 μmol/L. Brain complications encompassed cerebral infarction, stroke, cerebral hemorrhage, epilepsy, cerebral atrophy, Alzheimer’s disease, and cerebrovascular disease. Pulmonary complications included lung infections, atelectasis, tuberculosis, emphysema, pneumonia, pulmonary nodules, and lung cancer. ICU LOS refers to the duration of the initial ICU stay after surgery. The ICU discharge criteria included stable vital signs, adequate oxygenation, absence of active complications, normal or improved laboratory parameters, and a favorable multidisciplinary team assessment. Re-admission to ICU refers to re-admission to the ICU after the patient has been discharged to a normal ward or ambulatory care following the initial postoperative ICU stay. The postoperative information on pH, PaO₂, PaCO₂, K^+^, Na^+^, Ca^2+^, GLU, BE, Lac, Hb, and FiO₂ refers to the results of the first postoperative blood gas analysis.

### 2.3 Model endpoint definition

In this study, the endpoint was the occurrence of reintubation in patients with AAD undergoing surgical treatment.

### 2.4 Feature selection

The least absolute shrinkage and selection operator (LASSO) regression was employed to identify the most relevant features associated with reintubation risk. LASSO is a widely used machine-learning approach that integrates feature selection and model regularization by applying an L1 penalty, which forces the coefficients of less important variables to shrink to zero. This embedded feature selection method effectively reduces model complexity while retaining the most predictive variables (22). Importantly, LASSO regression is particularly advantageous in mitigating the impact of multicollinearity by penalizing correlated predictors and selecting the most informative features. In this study, LASSO regression was applied to the primary dataset, which included demographic characteristics, laboratory results, and perioperative information. The optimal penalty parameter (*λ*) was determined through 10-fold cross-validation, ensuring a balance between model performance and overfitting prevention. The final subset of selected features was subsequently incorporated into seven machine-learning models for predictive modeling.

### 2.5 Machine-learning models

In this study, seven distinct machine-learning models were employed for training, testing, and validation cohort, namely, multivariable logistics regression (MLR), decision-tree modeling (DT), random forest (RF), XGBoost (XGB), Support Vector Machines (SVM), k-nearest neighbors (kNN), and LightGBM (LGBM).

### 2.6 Performance evaluation

The predictive performance of the models was evaluated through discrimination and calibration. Discrimination was assessed using the area under the receiver operating characteristic (ROC) curve (AUC). Accuracy, sensitivity, specificity, precision, recall, and the F1-score were calculated to measure the accuracy of the model. To evaluate the accuracy and reliability of a model’s predictive probabilities, calibration was measured by calibration plots.

### 2.7 Model interpretation

To enhance the interpretability of the complex machine-learning models, we employed SHapley Additive exPlanation (SHAP) analysis to quantify the contribution of each feature to reintubation risk prediction. SHAP values provide both global interpretability, illustrating overall feature importance, and local interpretability, explaining how individual variables influence specific predictions. This method enables a comprehensive assessment of each predictor’s relative impact on model outputs, thereby improving the transparency and clinical applicability of the predictive model.

### 2.8 Statistical analysis

For statistical analyses, R version 4.2.1, Shiny version 0.5.1, and SPSS version 25.0 were utilized. Categorical and count data were presented as frequencies and percentages, and group comparisons were made using the chi-squared (*χ*^2^) test. Measurements that conformed to a normal distribution were expressed as mean ± standard deviation (SD), and *t*-tests were used for group comparisons. Non-normally distributed measurements are described using medians and interquartile ranges and were compared using the Mann–Whitney *U* test.

## 3 Results

### 3.1 Patient characteristics

**Figure 1:**
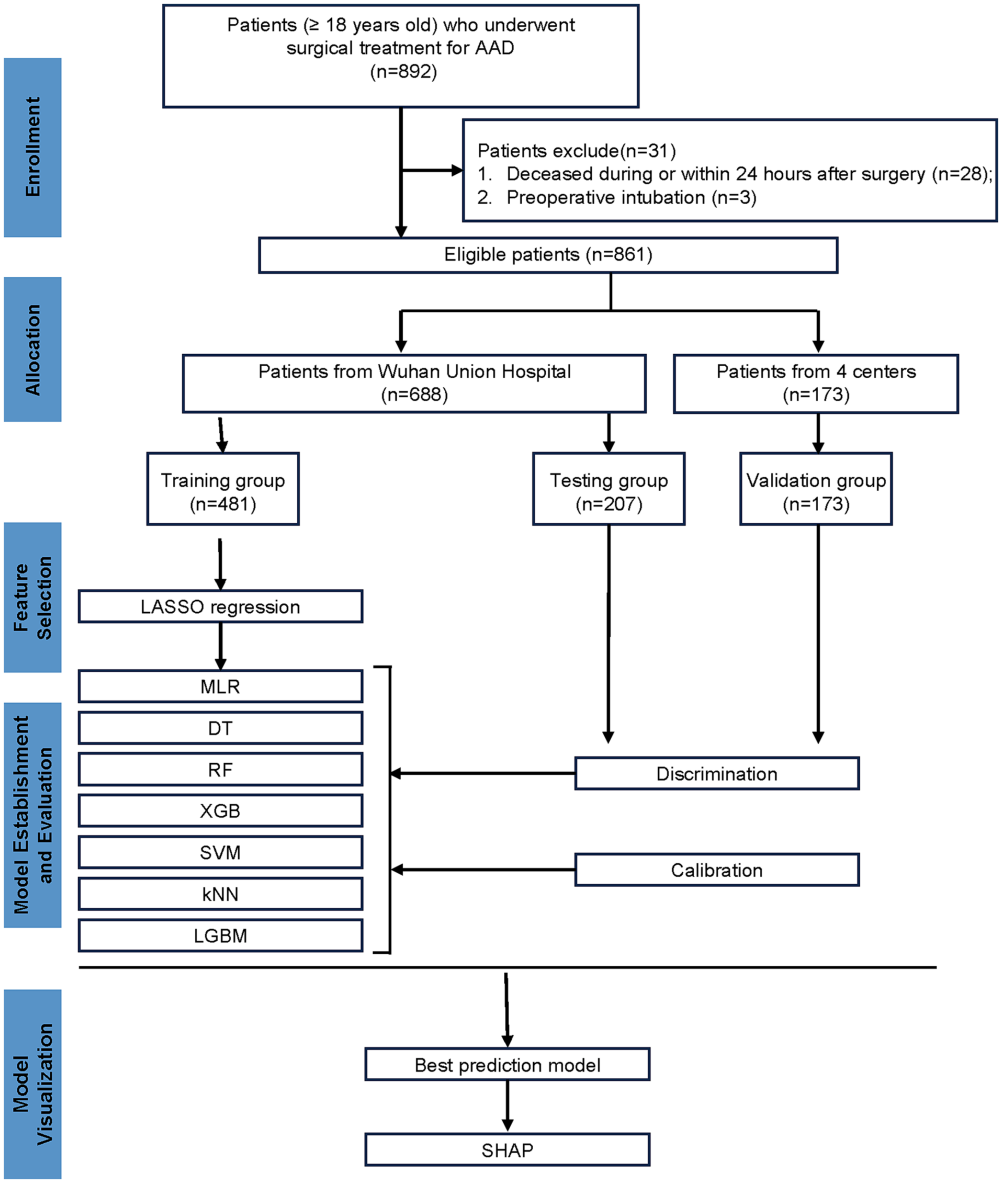
Flow chart of the study.

In accordance with the predefined inclusion and exclusion criteria, the study population underwent meticulous screening. Ultimately, a total of 861 patients were enrolled in this study (Figure 1), with 107 patients (12.43%) experiencing reintubation. The training, testing, and validation cohorts included 481, 207, and 173 patients with reintubation rates of 11.85, 13.04, and 13.29%, respectively. The mean (SD) age of the training, testing, and validation cohorts was 52.00 [43.00; 59.00] years, 53.00 [43.00; 59.00] years, and 52.00 [44.00; 58.00] years, respectively. The detailed characteristics are provided in Supplementary Table 1.

### 3.2 Influencing factors of reintubation

**Figure 2:**
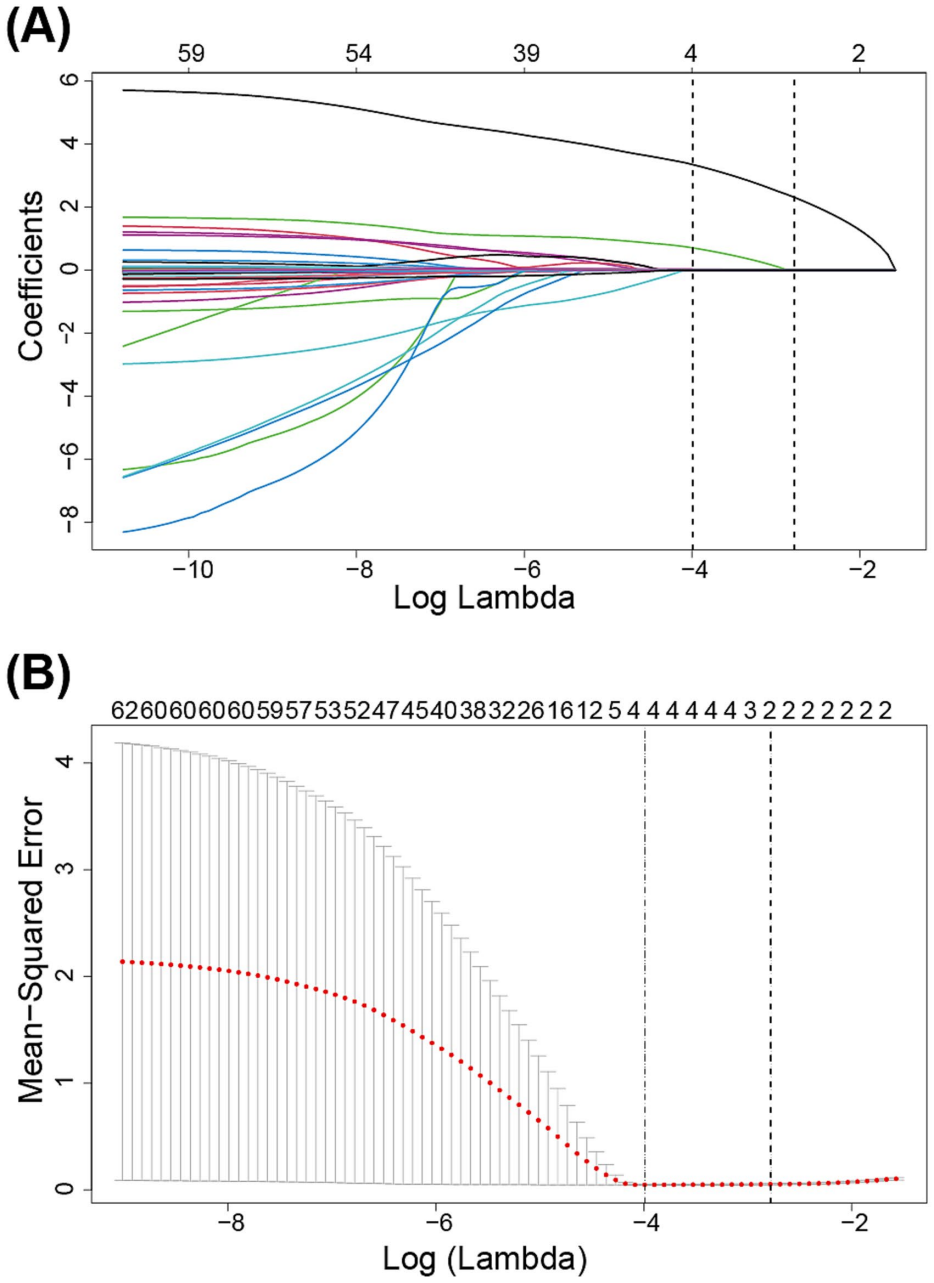
Variables were selected by the least absolute shrinkage and selection operator logistic regression model. **(A)** The least absolute shrinkage and selection operator coefficient profile plot. **(B)** 10-fold cross validation plot for the penalty term.

To ensure model accuracy and prevent overfitting, LASSO was utilized to select significant features associated with reintubation for subsequent modeling. The optimal parameters (lambda) in the LASSO model were chosen using 10-fold cross-validation (Figure 2). A vertical line is drawn at the values selected using 10-fold cross-validation, identifying four features with non-zero coefficients: re-admission to ICU, continuous renal replacement therapy (CRRT), ICU LOS, and duration of invasive mechanical ventilation (Table 1).

**Table 1:** The least absolute shrinkage and selection operator regression results of significant variables related to reintubation.

Variables	Coefficient	Lambda.min
ICU LOS	0.032609493	0.01847423
Re-admission to ICU	3.348052379	
Duration of invasive mechanical ventilation	0.007752884	
CRRT	0.706128965	

ICU, intensive care unit.

### 3.3 Development and assessment of risk prediction models

**Figure 3:**
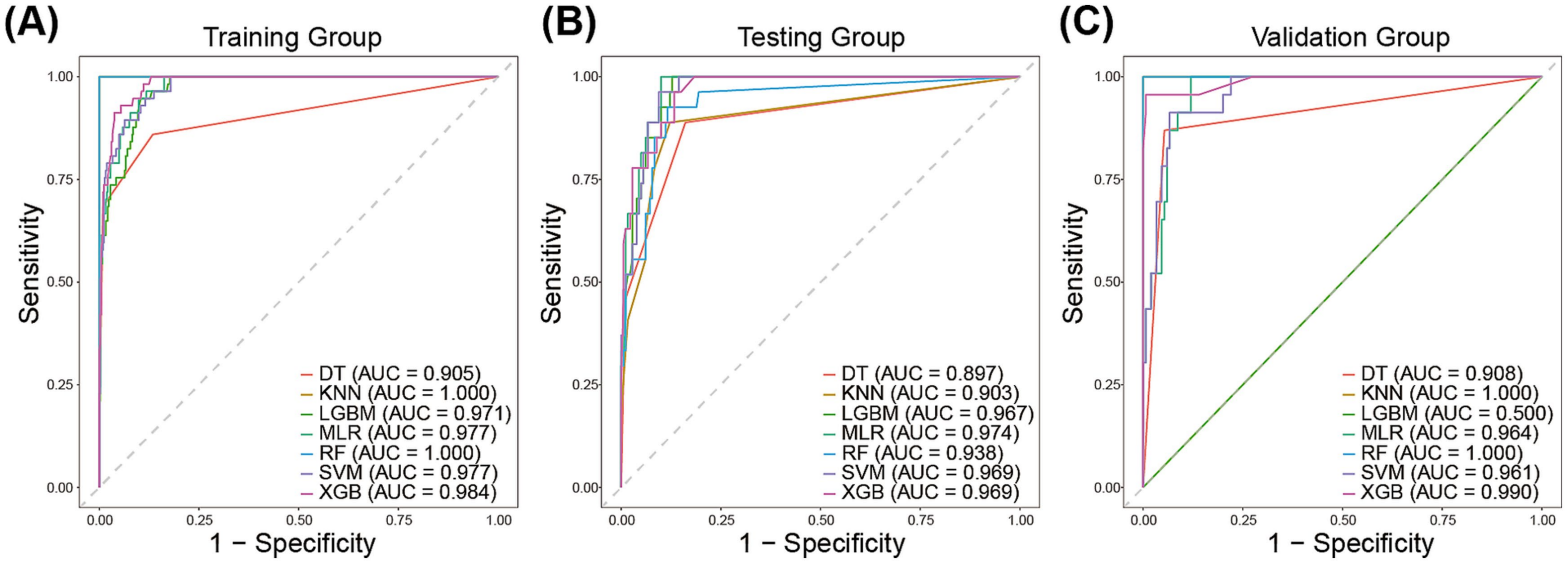
The area under the receiver operating characteristic curve of the prediction models in the training group **(A)**, the testing group **(B)**, and validation group **(C)**.

We employed seven machine learning algorithms to establish predictive models for reintubation and evaluated their predictive performance. Discrimination between the different models was quantified using the AUC of ROC (Figure 3). In the training group, the AUC values of different models ranged from 0.905 to 1.00, with the highest AUC achieved by the RF and kNN models (both AUC = 1.00) (Figure 3A). The AUC of all models in the testing group ranged between 0.897 and 0.974, followed by XGB and SVM (both AUC = 0.969) (Figure 3B). The kNN and XGB models exhibited the highest AUC in the validation group (AUC = 0.964) (Figure 3C).

Confusion matrices were generated for each model and compared using accuracy, precision, recall, and F1 score as metrics. The XGB model in the testing group achieved an accuracy of 0. 8,647, a precision of 0.4898, a recall of 0.8889, and an F1-score of 0.6316. The Accuracy, precision, recall, and F1-score of other models for the testing group are presented in Table 2. In addition, the XGB model achieved an accuracy of 0.9884, a precision of 0.9565, a recall of 0.9565, and an F1-score of 0.9565 in the validation group. Accuracy, precision, recall, and F1 scores of other models for the validation group are presented in Table 3.

**Table 2:** Evaluation metrics of seven models in the testing group.

Model	Accuracy	Sensitivity	Specificity	Precision	Recall	F1-score
MLR	0.9082	0.9630	0.9000	0.5909	0.9630	0.7324
DT	0.9227	0.9231	0.9227	0.4444	0.9231	0.6000
RF	0.9034	0.6296	0.9444	0.6296	0.6296	0.6296
XGB	0.8647	0.8889	0.8611	0.4898	0.8889	0.6316
SVM	0.0966	0.0370	0.1056	0.0062	0.0370	0.0106
kNN	0.8889	0.5556	0.9389	0.5556	0.5660	0.1304
LightGBM	0.8841	0.9259	0.8778	0.5319	0.9259	0.6757

MLR, multivariable logistics regression; DT, decision-tree modeling; RF, random forest; XGB, XGBoost; SVM, Support Vector Machines; kNN, k-nearest neighbors.

**Table 3:** Evaluation metrics of seven models in the validation group.

Model	Accuracy	Sensitivity	Specificity	Precision	Recall	F1-score
MLR	0.8960	1.0000	0.8800	0.5610	1.0000	0.7188
DT	0.9364	0.7143	0.9793	0.8696	0.7143	0.7843
RF	1.0000	1.0000	1.0000	1.0000	1.0000	1.0000
XGB	0.9884	0.9565	0.9933	0.9565	0.9565	0.9565
SVM	0.9306	0.9130	0.9333	0.6774	0.9130	0.7778
kNN	1.0000	1.0000	1.0000	1.0000	1.0000	1.0000
LightGBM	0.1329	1.0000	0.0000	0.1329	1.0000	0.2347

MLR, multivariable logistics regression; DT, decision-tree modeling; RF, random forest; XGB, XGBoost; SVM, Support Vector Machines; kNN, k-nearest neighbors.

Given the low AUC value of LGBM in the validation group, we subsequently show only the calibration plots of the six models other than LGBM in the training and test groups (Supplementary Figure 1). A model with good consistency is indicated by a curve that forms a 45° angle between the X-axis and the Y-axis. The calibration curve results showed that the XGB model exhibited good agreement between predicted and observed outcomes. Overall, the XGB model demonstrated good discrimination and calibration between the different groups.

### 3.4 Variable importance and variable interpretation

**Figure 4:**
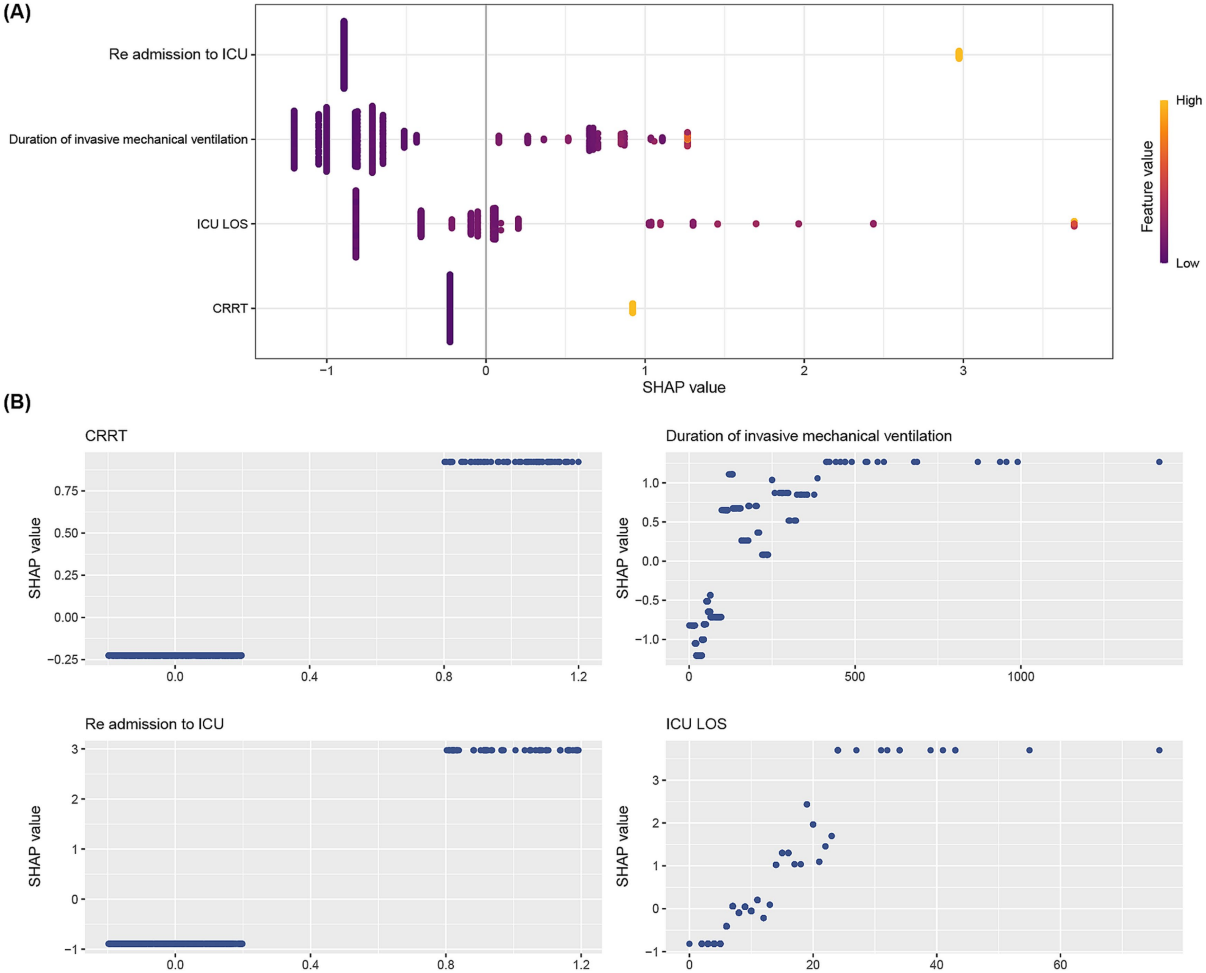
SHAP analyses of the XGB model for predicting reintubation. **(A)** Summary plot of SHAP values for each feature on the model output. The vertical axis ranks feature importance in descending order, with top features being most crucial to the model. The horizontal axis shows SHAP values, indicating if a feature increases or decreases the prediction. Yellow points mean higher observed values, while purple points mean lower ones. **(B)** SHAP dependence plot of the XGB model. Each panel shows how each feature impacts the Random XGB model’s predictions. The *x*-axis displays raw feature values, while the *y*-axis shows SHAP values. A SHAP value above zero for a feature suggests a higher risk of reintubation.

Given the above results, the XGB algorithm provided the fundamental framework for the ensemble learning models. We visualize the contribution of each feature to reintubation in the XGB model based on SHAP plots. Significant features were listed in descending order of their contribution to the model output in the SHAP summary plot. As shown in Figure 4A, the contributions were, from highest to lowest, re-admission to ICU, duration of invasive mechanical ventilation, ICU LOS, and CRRT. In addition, SHAP-dependent plots were employed to visualize the impact of individual features on the prediction of the risk of reintubation. When the SHAP value for a particular feature surpassed the zero threshold, it indicated an elevated risk of reintubation occurring. Conversely, a value below the zero threshold indicates a decreased risk of intubation (Figure 4B). Furthermore, we employed the feature influence diagram to illustrate prediction outcomes at the individual patient level. This tool offers a comprehensive depiction of the decision pathways used to generate predictions for individual patients, thereby enhancing our understanding of the factors contributing to the prediction of reintubation in certain patients (Supplementary Figures 2A,B) while others (Supplementary Figures 2C,D) are not predicted to develop the condition.

### 3.5 Construction of web calculator

**Figure 5:**
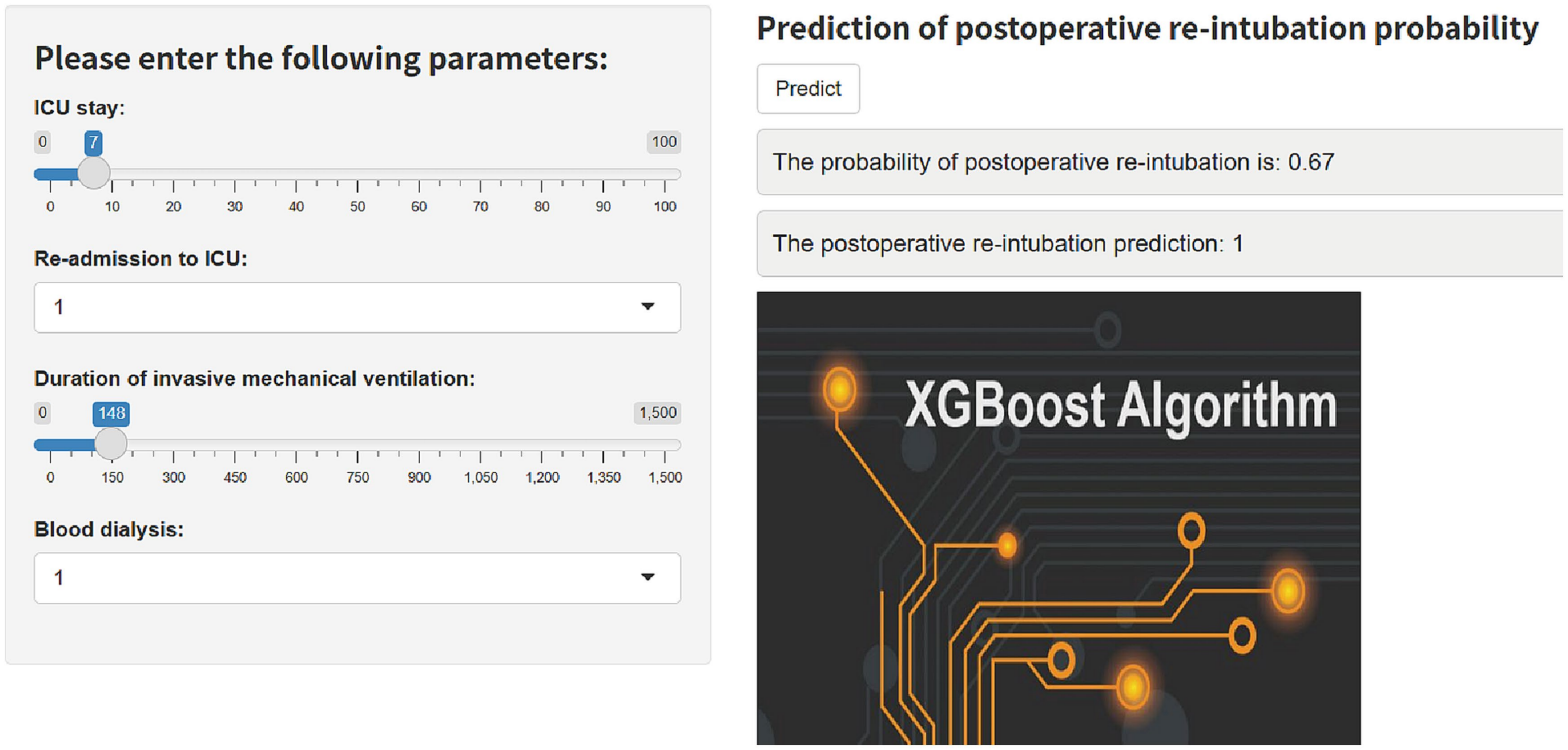
A web-based calculator for predicting postoperative reintubation in patients with AAD.

Based on the final machine learning model, we developed an easy-to-use web-based calculator that implements the risk equations for reintubation prediction (Figure 5), and this calculator is accessible at https://predict-xgb123.shinyapps.io/20241024/. Clinicians can utilize the calculator in real-time, allowing for rapid prediction of reintubation risk based on patient data.

## 4 Discussion

Early recognition of reintubation is crucial for improving the prognosis of patients with AAD and for preventing multiple postoperative adverse events. In this multicenter study, we have made several important contributions to the field. Firstly, we identified key laboratory and perioperative features associated with postoperative reintubation in AAD patients. Secondly, leveraging these variables, we developed and validated seven machine-learning models for predicting reintubation. Notably, the XGBoost (XGB) model demonstrated excellent performance in both the testing group and an external validation cohort, with strong discrimination and calibration abilities. To our knowledge, this is the first multicenter study to develop a predictive model for postoperative reintubation in AAD patients using machine-learning algorithms and multicenter data. These innovations allow for more accurate and timely detection of reintubation risk, enabling the development of personalized prevention strategies and enhancing early clinical decision-making. Our findings represent a significant step forward in leveraging advanced analytics to improve patient outcomes in AAD management.

Existing research on postoperative reintubation has prioritized risk factor identification while neglecting predictive validity assessments and clinical translation. Representative studies include a single-center retrospective analysis of 408 cardiac surgery patients linking arrhythmias and neurological dysfunction to reintubation risk (23), and an institutional analysis of 2,835 cases associating hemodynamic instability, dexmedetomidine dosing, and extubation timing with outcomes (9). A Chinese cohort of 492 AAD patients further developed a risk model incorporating age, smoking history, renal insufficiency, and intraoperative RBC transfusion (14). Despite these advances, two critical limitations persist—first, insufficient stratification by surgical type or pathology. Markedly heterogeneous reintubation rates across procedures (1.64% in CABG vs. 20.6% in thoracoabdominal aneurysm repair, with our AAD cohort at 12.43%) expose the inadequacy of generalized models for niche populations like AAD (14, 24, 25). Second, single-center datasets risk overfitting and geographical bias. Our study innovates by targeting the understudied high-risk AAD population, employing multicenter derivation/validation with robust sampling, and implementing interpretable machine learning (XGBoost with SHAP analysis) to quantify variable contributions. Comparative analysis with conventional scoring systems, including CARDOT score and SPORC score (26, 27), demonstrated our model’s superior predictive accuracy (higher AUC), validating its clinical utility for guiding AAD-specific extubation protocols and exemplifying the translational potential of machine learning in critical care workflows.

Our analysis identified ICU re-admission and prolonged initial ICU LOS as significant independent risk factors for postoperative reintubation in acute aortic dissection (AAD) patients. These parameters demonstrated particularly strong predictive value in our risk stratification model. This finding corroborates previous research by Ghali et al. (28), whose retrospective analysis of 610 ICU patients identified extended hospitalization duration as a critical risk factor for reintubation. The clinical implications of prolonged ICU LOS are substantial, as they are strongly associated with increased incidence of ICU-acquired complications including ventilator-associated pneumonia and bloodstream infections, which collectively elevate reintubation risk. Notably, our nomogram predictions align with existing literature reporting a 31% reintubation rate among ICU readmission cases (29). The pathophysiology underlying ICU readmission following cardiac surgery appears multifactorial, encompassing cardiopulmonary decompensation, deep sternal wound infections, critical limb ischemia, hemorrhagic complications, neurological sequelae, and systemic inflammatory responses (30, 31). Specific patient demographics – notably advanced age, end-stage renal disease, and chronic obstructive pulmonary disease – emerge as high-risk cohorts for ICU readmission (32, 33). These populations were frequently present with a diminished physiological reserve and multi-organ vulnerability, characteristics that synergistically amplify reintubation risk (8, 34, 35). While ICU readmission is a significant predictor of reintubation, its inclusion in the model may introduce circular reasoning. Future work should focus on identifying additional independent predictors and validating the model’s generalizability across diverse patient populations. Also, we recognize that this feature may not provide sufficient lead time for early intervention. To enhance the clinical utility of our model, future research should focus on the time dynamics between ICU readmission and reintubation. Specifically, we propose the development of a staged early warning mechanism to provide clinicians with a more nuanced understanding of the risk trajectory. Given the technical challenges and elevated complication rates associated with secondary intubation procedures (8), we emphasize the importance of vigilant respiratory monitoring and proactive pulmonary management in these vulnerable patients to mitigate reintubation necessity.

Our LASSO regression analysis identified CRRT as an independent predictor of postoperative reintubation in AAD patients. While direct evidence linking CRRT to reintubation remains limited, this association may be mediated through renal dysfunction markers—including acute kidney injury, elevated blood urea nitrogen, and creatinine levels—which are well-established contributors to respiratory complications (11, 36–38). Acheampong et al. (38) corroborated this relationship in their analysis of 8,809 surgical patients, identifying acute renal failure/dialysis as an independent reintubation risk factor. Similarly, Mendes et al. (39) demonstrated a dose-dependent association between preoperative creatinine elevation and reintubation likelihood, suggesting renal reserve as a critical determinant of postoperative respiratory stability. The pathophysiological interplay between CRRT and reintubation risk may involve dual mechanisms: impaired clearance of sedatives and neuromuscular blockers in renal insufficiency, prolonging drug-induced respiratory depression (40), and fluid overload secondary to oliguria/anuria, predisposing to pulmonary interstitial edema, reduced lung compliance, and airway obstruction (41). These mechanisms collectively heighten vulnerability to respiratory failure despite mechanical ventilation weaning. Given the clinical significance of this relationship, future large-scale prospective studies are warranted to delineate causal pathways and optimize perioperative management strategies in this high-risk cohort.

Our analysis identified prolonged invasive mechanical ventilation duration as a significant independent predictor of reintubation in AAD patients, consistent with prior research (42). While early extubation as part of fast-track anesthesia protocols has demonstrated safety without elevating reintubation risk (43), extended invasive mechanical ventilation duration correlates strongly with adverse outcomes. Protracted endotracheal intubation compromises respiratory mucosal integrity, impairs mucociliary clearance, and predisposes patients to ventilator-associated pneumonia and atelectasis, thereby amplifying reintubation likelihood (44, 45). Notably, delayed extubation may mask early neurological deficits, including stroke manifestations, by limiting clinical assessment of consciousness and motor function. This diagnostic obscuration can delay neuroprotective interventions, indirectly exacerbating respiratory compromise and necessitating reintubation (46). Therefore, it is suggested to remove endotracheal intubation and switch to spontaneous breathing as soon as conditions permit (42). Given these risks, meticulous respiratory monitoring and proactive extubation planning are imperative for AAD patients requiring prolonged invasive mechanical ventilation to mitigate secondary respiratory failure and its associated morbidity.

Machine learning has emerged as a valuable tool for clinical decision-making, capable of handling high-dimensional and complex datasets by integrating multiple data sources to build a statistical model that predicts a specific outcome (47, 48). The choice of different machine learning algorithms has a significant effect on the predictive accuracy of the model, thus the selection of the appropriate algorithm to build the predictive model is crucial. In this study, we compared XGB models with six other machine learning models to develop the most accurate predictive model for postoperative reintubation in patients with AAD. Furthermore, many models and statistical methods involve complex mathematical terms and formulas, which can be challenging for healthcare professionals to interpret. To use this model in daily scenarios and to deepen the understanding of the risk factors in the model, we employed SHAP to visualize the XGB model. SHAP stands out among the many visualization techniques for its ability to consider the effects of individual features and the potential synergistic effects of groups of variables on the overall model. To address this issue, in this study, the SHAP method was used to interpret the contribution of individual predictors to the predictive model, further deepening the understanding of reintubation risk factors. On the other hand, to promote the widespread application of our model, we have developed a user-friendly online prediction platform for cardiac surgeons, enabling them to easily identify patients at high risk of reintubation. This platform can be seamlessly integrated into electronic medical record systems, which are increasingly used in clinical settings. With the integration of machine learning models, clinicians can benefit from automated reintubation risk assessments, eliminating the need for manual calculations. The system would capture key clinical features from the patient’s electronic medical record, providing timely warnings to clinicians through computer or mobile devices. This would enable more proactive patient management, reducing the risk of adverse outcomes.

This study has several limitations that warrant consideration. First, as an observational and retrospective cohort study, our findings may be subject to biases arising from unmeasured confounders. Specifically, the lack of detailed data on pulmonary and cerebral disease may limit the generalizability of our risk stratification model. Additionally, postoperative assessments were inconsistently documented across centers, which may have led to the omission of unrecognized risk factors. While these limitations cannot be fully overcome due to the inherently absent data, we attempted to mitigate their impact by employing robust statistical methods based on multiple machine-learning algorithms. Second, our findings may not be generalizable beyond the participating centers, all of which are from a single country. Future studies should include more diverse cohorts. Finally, we acknowledge the potential for multicollinearity among predictors, which could affect model interpretability. Future work should explore methods to detect and address multicollinearity, such as variance inflation factors or principal component analysis, to further enhance model interpretability.

## 5 Conclusion

Taken together, our study identified a set of novel predictors and developed predictive models for postoperative reintubation in patients with AAD. We are confident that the refined risk prediction model will serve as a valuable tool for surgeons, aiding in the anticipation of reintubation risk and the formulation of preemptive, tailored intervention strategies. This advancement is expected to broaden the scope and applicability of risk prediction models within clinical practice, ultimately contributing to improved patient outcomes.

## Data Availability

The raw data supporting the conclusions of this article will be made available by the authors, without undue reservation.
