# Supplementary material for: Multiple automated machine-learning prediction models for postoperative reintubation in patients with acute aortic dissection: a multicenter cohort study
Source: Front Med (Lausanne). 2025 Apr 11;12:1531094. doi: 10.3389/fmed.2025.1531094 (PMC12021851; doi:10.3389/fmed.2025.1531094)
Supplement: Supplementary file 1 [file Data_Sheet_1.docx]

Supplementary Material

# Supplementary Tables

**Supplementary TABLE 1** Demographic characteristics of the non-reintubation and reintubation group.

| Variates | Total (n=861) | Non-reintubation (n=754) | Reintubation (n=107) | P value |
| --- | --- | --- | --- | --- |
| Age (yr) | 52.00 [43.00;59.00] | 52.00 [43.00;59.00] | 53.00 [44.00;58.50] | 0.681 |
| Gender |  |  |  | 0.263 |
| Female | 163 (18.93%) | 138 (18.30%) | 25 (23.36%) |  |
| Male | 698 (81.07%) | 616 (81.70%) | 82 (76.64%) |  |
| Height (m) | 1.70 [1.66;1.74] | 1.70 [1.67;1.74] | 1.70 [1.65;1.73] | 0.514 |
| Weight (Kg) | 74.00 [65.00;80.00] | 74.00 [65.00;80.00] | 70.00 [63.50;81.50] | 0.512 |
| BMI (kg/m^2^) | 25.35 [23.31;27.68] | 25.35 [23.31;27.68] | 25.35 [23.24;27.44] | 0.493 |
| **Medical history** |  |  |  |  |
| Pulmonary.Complications | 102 (11.85%) | 87 (11.54%) | 15 (14.02%) | 0.560 |
| Hypertension |  |  |  | 0.062 |
| 0 | 259 (30.08%) | 225 (29.84%) | 34 (31.78%) |  |
| 1 | 106 (12.31%) | 95 (12.60%) | 11 (10.28%) |  |
| 2 | 69 (8.01%) | 67 (8.89%) | 2 (1.87%) |  |
| 3 | 427 (49.59%) | 367 (48.67%) | 60 (56.07%) |  |
| Diabetes mellitus | 50 (5.81%) | 47 (6.23%) | 3 (2.80%) | 0.231 |
| Marfan syndrome | 12 (1.39%) | 10 (1.33%) | 2 (1.87%) | 0.652 |
| Smoking | 287 (33.33%) | 250 (33.16%) | 37 (34.58%) | 0.855 |
| Alcohol | 216 (25.09%) | 186 (24.67%) | 30 (28.04%) | 0.527 |
| Brain complications | 55 (6.39%) | 46 (6.10%) | 9 (8.41%) | 0.482 |
| Renal insufficiency | 32 (3.72%) | 26 (3.45%) | 6 (5.61%) | 0.272 |
| Previous aortic valve surgery | 18 (2.09%) | 16 (2.12%) | 2 (1.87%) | 1.000 |
| Previous total aortic arch replacement | 11 (1.28%) | 10 (1.33%) | 1 (0.93%) | 1.000 |
| Previous endovascular treatment | 20 (2.32%) | 20 (2.65%) | 0 (0.00%) | 0.159 |
| **Preoperative test** |  |  |  |  |
| Myoglobin (ng/mL) | 53.15 [53.15;53.15] | 53.15 [53.15;53.15] | 53.15 [53.15;53.15] | 0.999 |
| CK MB (U/L) | 0.80 [0.80;0.80] | 0.80 [0.80;0.80] | 0.80 [0.80;0.80] | 0.576 |
| Hypersensitive troponin T (ng/L) | 8.80 [8.80;8.80] | 8.80 [8.80;8.88] | 8.80 [8.20;8.80] | 0.744 |
| RBC count (×10^12^/L) | 4.12 [4.12;4.12] | 4.12 [4.12;4.12] | 4.12 [4.12;4.12] | 0.732 |
| Hemoglobin (g/L) | 126.00 [126.00;126.00] | 126.00 [126.00;126.00] | 126.00 [126.00;126.00] | 0.484 |
| WBC count (×10^9^/L) | 9.67 [9.67;9.67] | 9.67 [9.67;9.67] | 9.67 [9.67;9.67] | 0.251 |
| Percentage of neutrophils (%) | 81.00 [81.00;81.00] | 81.00 [81.00;81.00] | 81.00 [81.00;81.00] | 0.465 |
| Platelet count (×10^9^/L) | 158.00 [158.00;158.00] | 158.00 [158.00;158.00] | 158.00 [158.00;158.00] | 0.526 |
| ALT (U/L) | 25.00 [25.00;25.00] | 25.00 [25.00;25.00] | 25.00 [25.00;25.00] | 0.106 |
| AST (U/L) | 25.00 [25.00;25.00] | 25.00 [25.00;25.00] | 25.00 [25.00;25.00] | 0.006 |
| Total serum bilirubin (umol/L) | 16.80 [16.80;16.80] | 16.80 [16.80;16.80] | 16.80 [16.80;16.80] | 0.671 |
| Direct serum bilirubin (umol/L) | 5.45 [5.45;5.45] | 5.45 [5.45;5.45] | 5.45 [5.45;5.45] | 0.989 |
| Creatinine (umol/L) | 81.80 [81.80;81.80] | 81.80 [81.80;81.80] | 81.80 [81.80;81.80] | 0.021 |
| Blood urea nitrogen (umol/L) | 6.48 [6.48;6.48] | 6.48 [6.48;6.48] | 6.48 [6.48;6.48] | 0.141 |
| Serum uric acid (umol/L) | 382.55 [382.55;382.55] | 382.55 [382.55;382.55] | 382.55 [382.55;382.55] | 0.042 |
| Albumin (g/L) | 37.40 [37.40;37.40] | 37.40 [37.40;37.40] | 37.40 [37.40;37.40] | 0.341 |
| Glomerular filtration rate (ml/min/1.703m^2^) | 90.59 [90.59;90.59] | 90.59 [90.59;90.59] | 90.59 [90.59;90.59] | 0.025 |
| D-dimer (mg/L) | 5.10 [5.10;5.10] | 5.10 [5.10;5.10] | 5.10 [5.10;5.10] | 0.118 |
| FDP (mg/L) | 17.20 [17.20;17.20] | 17.20 [17.20;17.20] | 17.20 [17.20;17.20] | 0.215 |
| PT (s) | 14.00 [14.00;14.00] | 14.00 [14.00;14.00] | 14.00 [14.00;14.00] | 0.307 |
| APTT (s) | 37.10 [37.10;37.10] | 37.10 [37.10;37.10] | 37.10 [37.10;37.10] | 0.550 |
| INR | 1.11 [1.11;1.11] | 1.11 [1.11;1.11] | 1.11 [1.11;1.11] | 0.266 |
| EF (%) | 62.00 [62.00;62.00] | 62.00 [62.00;62.00] | 62.00 [62.00;62.00] | 0.717 |
| Diameter of aortic sinus (mm) | 41.00 [41.00;41.00] | 41.00 [41.00;41.00] | 41.00 [41.00;41.00] | 0.190 |
| Diameter of ascending aorta (mm) | 44.00 [43.00;44.00] | 44.00 [43.00;44.00] | 44.00 [44.00;45.00] | 0.502 |
| **Intraoperative information** |  |  |  |  |
| RBC transfusion (U) | 2.00 [0.00;4.00] | 2.00 [0.00;4.00] | 3.00 [0.50;6.00] | 0.054 |
| Plasma transfusion (U) | 0.00 [0.00;400.00] | 0.00 [0.00;400.00] | 0.00 [0.00;400.00] | 0.962 |
| Platelet transfusion (U) | 1.00 [0.00;2.00] | 1.00 [0.00;2.00] | 2.00 [1.00;2.50] | 0.022 |
| CPB duration (min) | 184.00 [157.00;225.00] | 184.00 [156.00;223.00] | 197.00 [165.00;253.50] | 0.004 |
| Aortic clamping duration (min) | 107.00 [91.00;134.00] | 107.00 [90.00;132.00] | 117.00 [95.00;146.50] | 0.008 |
| **Postoperative information** |  |  |  |  |
| Intra-aortic balloon pump | 5 (0.58%) | 5 (0.66%) | 0 (0.00%) | 1.000 |
| ICU LOS (Day) | 6.00 [5.00;9.00] | 6.00 [5.00;7.75] | 15.00 [8.00;24.00] | <0.001 |
| Re admission to ICU | 78 (9.06%) | 26 (3.45%) | 52 (48.60%) | <0.001 |
| Duration of invasive mechanical ventilation (h) | 53.00 [35.00;92.00] | 45.00 [32.00;70.00] | 250.00 [149.00;482.00] | <0.001 |
| CRRT, n (%) | 107 (12.43%) | 64 (8.49%) | 43 (40.19%) | <0.001 |
| PH | 7.44 [7.42;7.45] | 7.44 [7.43;7.46] | 7.44 [7.40;7.44] | 0.012 |
| PO2 | 100.00 [86.80;118.00] | 100.00 [89.50;120.00] | 100.00 [73.25;114.00] | 0.062 |
| PCO2 | 36.00 [34.60;37.40] | 36.00 [34.60;37.10] | 36.00 [35.30;38.25] | 0.154 |
| K^+^ | 4.20 [4.10;4.40] | 4.20 [4.10;4.40] | 4.20 [4.00;4.30] | 0.342 |
| Na^+^ | 144.00 [143.00;144.00] | 144.00 [143.00;144.00] | 144.00 [143.00;145.00] | 0.622 |
| Ca2^+^ | 1.14 [1.12;1.16] | 1.14 [1.12;1.16] | 1.14 [1.12;1.16] | 0.627 |
| GLU | 10.80 [10.30;11.50] | 10.80 [10.20;11.40] | 10.80 [10.65;12.15] | 0.176 |
| BE | 0.50 [-0.10;1.20] | 0.50 [0.10;1.20] | 0.50 [-1.45;0.50] | 0.003 |
| Lac | 2.70 [2.40;3.30] | 2.70 [2.30;3.10] | 2.70 [2.70;5.35] | <0.001 |
| Hb | 9.90 [9.50;10.20] | 9.90 [9.70;10.30] | 9.90 [8.95;10.00] | 0.031 |
| FiaO2 | 1.00 [1.00;1.00] | 1.00 [1.00;1.00] | 1.00 [1.00;1.00] | 0.902 |

BMI, body mass index; ICU, intensive care unit; CRRT, continuous renal replacement therapy; ICU LOS, length of stay in the intensive care unit; RBC, red blood cells; WBC, white blood cells; ALT, alanine transaminase; AST, glutamic oxalacetic transaminase; FDP, fibrin degradation products; PT, prothrombin time; APTT, activated partial prothrombin time; INR, international normalized ratio; EF, ejection fraction; CPB, cardiopulmonary bypass.

# Supplementary Figures

**
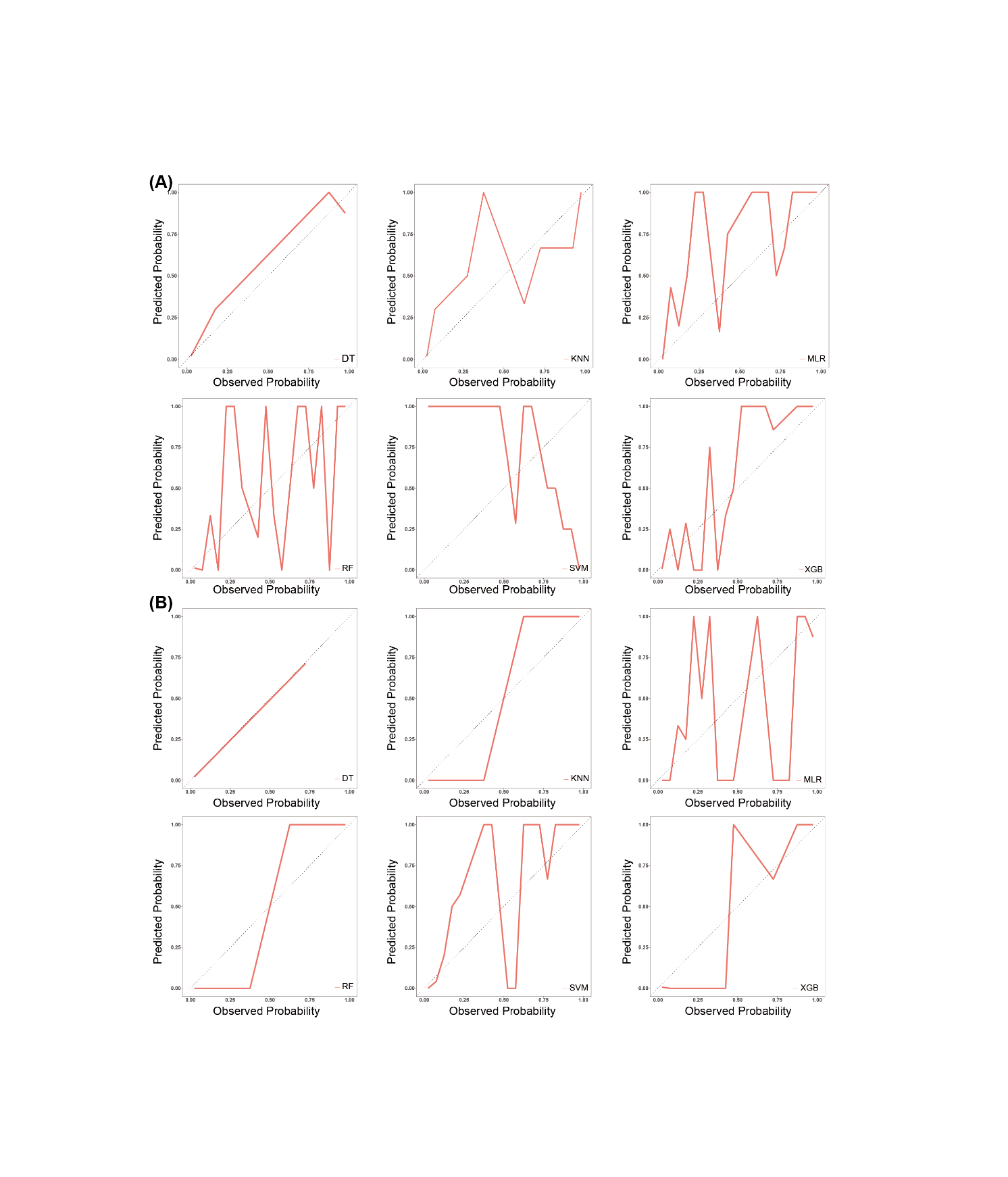
**

**Supplementary Figure 1.** Calibration plot of the prediction models in the testing group **(A)** and the validation group **(B)**.


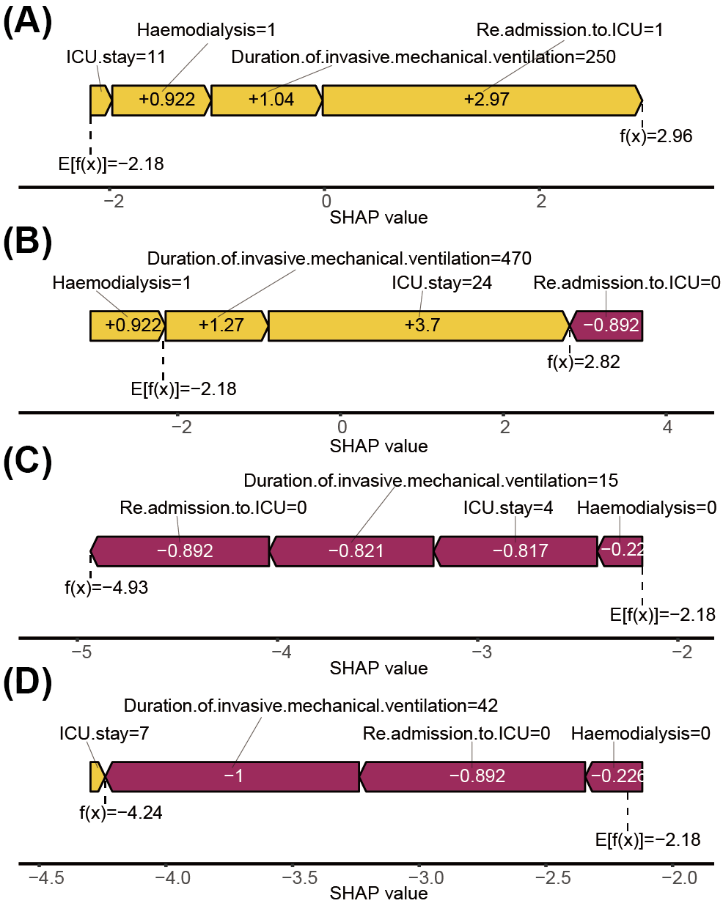


**Supplementary Figure 2.** Feature influence diagram of individual patient based on the SHAP decision analysis. **(A)-(B)** illustrate three patients predicted as reintubation while **(C)-(D)** show examples of three patients predicted as non-reintubation
