# Supplementary figures and images for: Multiple automated machine-learning prediction models for postoperative reintubation in patients with acute aortic dissection: a multicenter cohort study
Source: Front Med (Lausanne). 2025 Apr 11;12:1531094. doi: 10.3389/fmed.2025.1531094 (PMC12021851; doi:10.3389/fmed.2025.1531094)

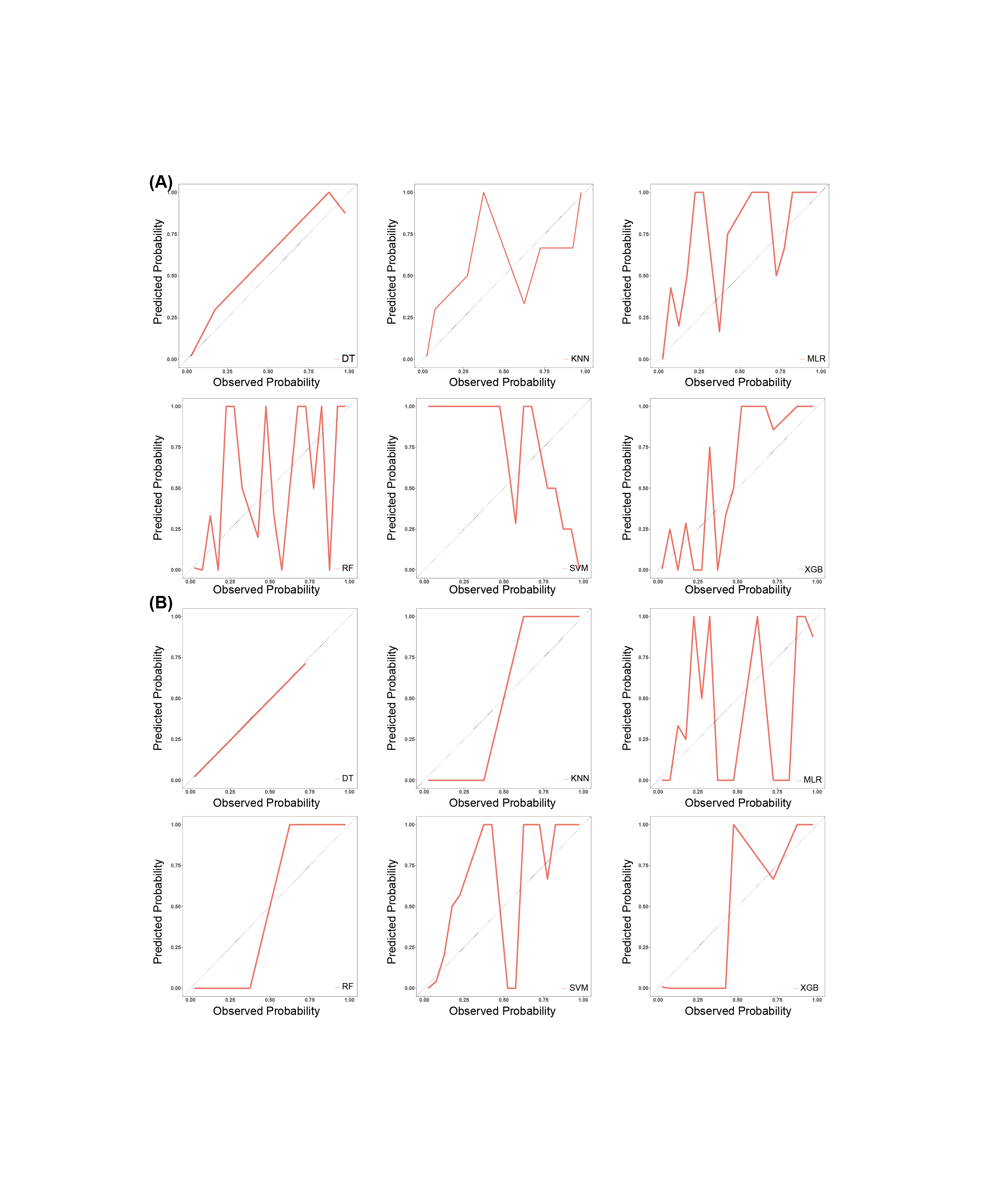

Supplement: Supplementary file 2 [file Image_1.tif]

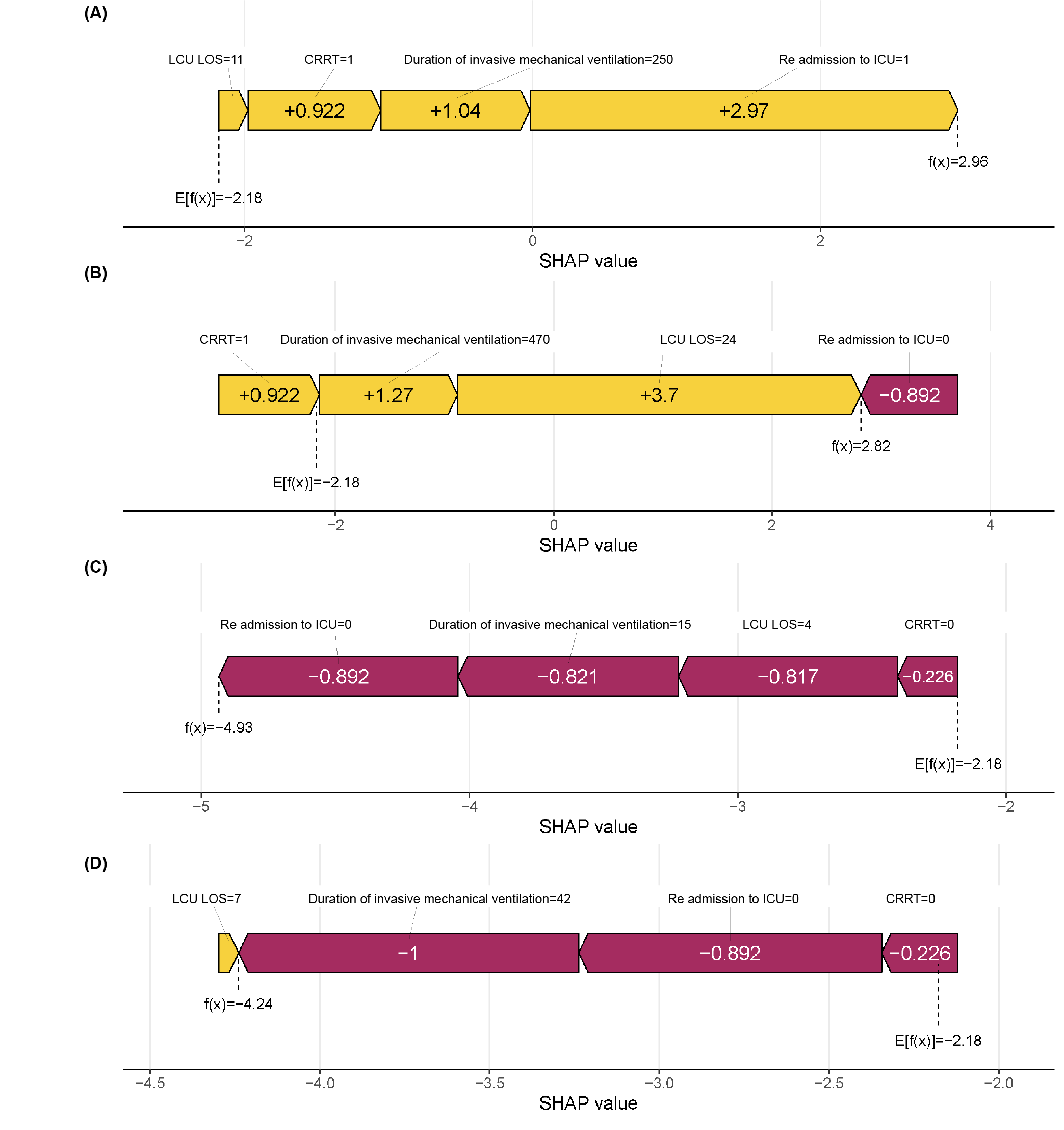

Supplement: Supplementary file 3 [file Image_2.tif]
